# Rheumatoid arthritis classification using hybrid pretrained quantum convolutional neural network

**DOI:** 10.3389/frai.2026.1794495

**Published:** 2026-08-19

**Authors:** D. Jeyamani, Rukmani Panjanathan

**Affiliations:** School of Computer Science and Engineering, Vellore Institute of Technology-Chennai Campus, Chennai, India

**Keywords:** activation functions, convolutional neural networks, feature extraction, hyperparameter tuning, medical imaging, quantum computing, quantum convolutional neural network, rheumatoid arthritis classification

## Abstract

Rheumatoid arthritis (RA) is a chronic autoimmune disease where early diagnosis is critical for preventing irreversible joint damage. Recent advances in quantum computing have established potential advantages in modelling complex, high-dimensional biomedical data. The main objective of the work is to propose a Hybrid Pretrained Quantum Convolutional Neural Network (HP-QCNN) for automated RA classification from hand X-ray images. The proposed framework integrates pretrained convolutional neural networks with a variational quantum layer to enhance feature representation and classification performance. The hybrid architecture employs a 4-qubit quantum layer based on variational quantum circuits, utilizing quantum superposition and entanglement to perform parameter-efficient feature transformations. This quantum layer is embedded between a pretrained feature extractor and a classical classification head. Multiple pretrained backbones were explored in a hybrid quantum-optimized activation function. The proposed VGG16-based HP-QCNN achieved the highest classification accuracy, outperforming DenseNet121, MobileNetV2, EfficientNetB3, and InceptionV3. The proposed model highlights quantum computing efficiency by achieving an accuracy as 92.41%, the highest ROC-AUC, and specificity over 95%, which outperforms the classical VGG16 baseline by 7.3, 3, and 10%, respectively. Stratification ensures the class distribution, while weighted classes reduce the residual class imbalance. The best Matthews correlation coefficient and Cohen’s kappa score, which are above the baseline (0.7), indicate strong class separability and reliability under class imbalance. These results confirm the effectiveness of quantum-enhanced deep learning in medical image analysis.

## 1 Introduction

Approximately 1.71 billion people globally are affected by musculoskeletal disorders, with over 18 million suffering from rheumatoid arthritis (RA). The global prevalence of RA is approximately 0.35%, which is also reflected in India. There are 26.9 deaths per 100,000 person-years among RA patients, which is higher than the 18.9 deaths per 100,000 person-years in the general population. In 2020, around 18 million people were affected by RA, and projections indicate this could rise to 31.7 million by 2050, an 80% increase (Black et al., 2023). This autoimmune condition usually occurs in individuals over the age of 40 and is more common in women than in men (Chodara et al., 2017; Kay and Upchurch, 2012). Rheumatoid arthritis (RA) can affect various joints, including hand joints, wrists, elbows, knees, ankles, and metatarsophalangeal (MTP) in the feet. Rheumatoid arthritis can be diagnosed using the Disease Activity Score, modified Sharp van der Heijde score (SvH) (van der Heijde, 2000), and the “American College of Rheumatology (ACR) and European League Against Rheumatism (EULAR)-2010” classification criteria (Kay and Upchurch, 2012). Hand X-rays remain an important standard for finding RA progression by grades 0–4, but radiologist inter-observer variability (*κ*) stands between 0.4 and 0.7. So subtle changes are not detected in an earlier manner (Mate et al., 2021; Wang et al., 2022). Automated detection from hand radiographs addresses this gap, enabling preventive intervention to prevent irreversible joint damage.

Deep learning models have proven effectiveness in image recognition and classification tasks, contributing to the creation of more resilient models for medical imaging. The digitalization of medical records has enabled the utilization of deep learning in computer vision tasks related to a range of medical images, including X-ray, ultrasound, CT, and MRI, which benefit from these advancements. Convolutional neural networks (CNNs) have emerged as the preferred method for algorithmic feature extraction in medical image analysis (Avramidis et al., 2021). In studies focused on the diagnosis of rheumatoid arthritis (RA), two principal data modalities are predominantly utilized: clinical data and medical imaging data. For the automated classification and analysis of these data, a range of deep learning approaches, including artificial neural networks (ANNs), convolutional neural networks (CNNs), deep CNN architectures, recurrent neural networks (RNNs), and Long Short-Term Memory (LSTM)-based models, have been extensively adopted (Hügle et al., 2020).

The detection ability of the severity of chronic musculoskeletal disorders from medical images is essential for early intervention, such as the treatment of rheumatoid arthritis (RA), where delayed diagnosis can have irreversible effects on the joints (Yan et al., 2024). Radiographic data often reveals subtle structural changes and high-dimensional correlations that conventional deep learning models cannot capture. As a result of recent advances in quantum machine learning (QML), variational quantum circuits can insert classical features in high-dimensional Hilbert spaces, resulting in a more expressive representation with fewer parameters that must be trained (Schuld and Killoran, 2019). The QML modelling approach utilises quantum superposition and entanglement to capture complex features that cannot be captured by conventional methods. In medical image classification tasks, such as musculoskeletal imaging, hybrid quantum-classical frameworks with pretrained convolutional neural networks showed improved performance (Senokosov et al., 2024). As a result of these findings, hybrid quantum-classical architectures are being adopted for RA classification to represent features better and to provide strong clinical decision support with the following objectives.

- To develop a hybrid pretrained Quantum model for the classification of Rheumatoid Arthritis based on Hand X-ray images.
- To evaluate the accuracy as well as the effectiveness of this model in determining RA and Non-RA using hand X-ray images.
- To investigate the effectiveness of activation functions that enhance gradient flow and learning efficiency of the proposed model in Hand Rheumatoid Arthritis classification.
- To conduct a comparative analysis with different hybrid quantum combinations that use different pretrained models with different activation functions.

The remaining sections of this work have been structured as follows. Section II gives an overview of the existing methodologies that were used to classify rheumatoid arthritis. Section III describes the proposed method that was used in the present study. The planned pretrained Hybrid Experimental setup is discussed in the following section. In the end, Sections V and VI detailed the work with important findings, conclusions, and suggestions for the future, respectively.

## 2 Literature review

The following Table 1 provides a summary of classical CNNs, Pretrained CNNs, and other models that were utilized for Rheumatoid arthritis classification. Table 2 gives a summary of Quantum/Hybrid RA & Medical Imaging Studies.

**Table 1:** Classical CNN-based RA hand x-ray studies.

Author(s)	Dataset	Methodology	Key Metrics	Limitations
Izumi et al. (2023)	216 hand X-rays	ResNet/DenseNet/ViT, 5-fold CV	Accuracy 97%, AUC92%	Small dataset, foot joint complexity, no, multi-centre validation
Wang et al. (2022)	3,818 X-rays	ResNet-Dwise50 depthwise convolution	MAE14.90, RMSE 22.01	Scoring imbalance, high computational demands, subjective annotations
Peng et al. (2024)	344 radiographs	GoogLeNet/VGG16/AlexNet	GoogLeNet AUC 97.8%, VGG16 83.4%	Subtle stage differences, limited annotations, single-region data
Üreten and Maraş (2022)	Kırıkkale Univ X-rays	YOLOv4+VGG16 transfer	RA vs. normal 90.7%, overall 80.6%	Single-centre bias, no multi-modal data
Honda et al. (2023)	1,503 INDRA X-rays	CNN for SvH erosion/JSN	ICC 0.98, AUC 0.75	No public validation dataset, generalization challenges
Rohrbach et al. (2019)	102 K finger joints	CNN+VGG16 transfer	Balanced_ACC 47.5%, ±1 ACC 83%	Severe imbalance, label noise, limited global accuracy
Langs et al. (2009).	20 RA patients	ASMs + classifiers	JSW diff 0.08–0.25 mm	Incomplete mTSS, imaging protocol dependency
Huo et al. (2016)	104 X-rays (52 pts)	Active contour JSW	JSW error 6.8%	Hand positioning variability, excluded joints
Rajesh et al. (2024)	Hand radiographs	Threshold + GLCM	Precision 85%, F1 82%	Computational demands, limited dataset details

**Table 2:** Quantum-hybrid RA & medical imaging studies.

Author(s)	Dataset	Methodology	Key metrics	Limitations
Cai et al. (2025)	1,655 hand radiographs	Custom model -VGG 8	AUC 81% Acc. 74%	External validation drops
Ajlouni et al. (2023)	RENBRANDT medical images dataset	Adaptive HQ-CNN + PQC	Val acc 98%	Brain tumours only, no musculoskeletal
Kesavapillai et al. (2024)	X-ray + thermal	CNN + QSVM	90% X-ray, 93.75% thermal	Dual-modality required, complex pipeline
Ahalya et al. (2023)	1,440 thermal hand images	CNN + PennyLane QNN	RANet 95%, QNN 93.3%	Thermal only (no X-ray), dataset-specific

CNNs for RA hand X-ray analysis performed well, with AUC ranging from 83 to 97% and balanced accuracy up to 83% across a wide range of datasets from 20 to 102 K joints. The MAE for RA scoring was 14.90, and the ICC for erosion detection was 0.98 when ResNet variants and transfer learning were used. However, small datasets (*n* < 400), single-centre bias, severe class imbalance, and lack of multi-centre validation consistently limit clinical translation.

Quantum-enhanced methodologies have given 93–97% accuracy primarily on hand thermal images using Quantum Neural Networks and Quantum Support Vector Machines, with 2/3 training epochs when compared with classical CNNs. RANet with SVM reached 97% on 1,440 thermal images, while RA-XTNet combines X-ray thermal for 90–93.75%. Limitations include thermal-only focus, dual-modality requirements, and lack of X-ray-specific quantum optimization for RA. Existing quantum RA models performed well on thermal imaging, with accuracy ranging between 93 and 97%, but underexplored hand X-ray mTSS scoring, whereas classical CNNs and pretrained CNNs performed at 83–97% due to imbalance and generalization limits. Existing RA imaging studies are mostly based on classical CNNs or segmentation/measurement pipelines, and even though they achieve good results, they face challenges such as data imbalance and label noise, as well as the computational limitations of classical convolutions when capturing subtle, nonlinear radiographic patterns. In addition, QML research has shown that hybrid quantum–classical architectures, such as HQCNNs, QCNNs, and quanvolutional models, can outperform or match strong CNN baselines with fewer parameters and faster convergence, particularly when interacting with complex features. Quantum-enhanced algorithms have mainly been applied to brain tumours (Sriya et al., 2023), skin lesions (Reka et al., 2024) COVID-19 and RA thermal images (Kesavapillai et al., 2024), which shows that any prior work did not integrate variational quantum layers into pretrained CNNs for RA hand X-ray classification. This gap presents a unique opportunity to explore how variational quantum layers can enhance RA classification by capturing subtle patterns, resolving data challenges, and improving efficiency, leading to medical imaging innovations using hybrid quantum-classical models. This work fills this gap through an HP-QCNN architecture, achieving 92.41% accuracy on X-ray datasets while requiring no thermal preprocessing or dual-modality pipelines. Although the quantum method explored qubits, gates, and multi-activation features while classifying RA and Healthy.

## 3 Proposed method

**Figure 1:**
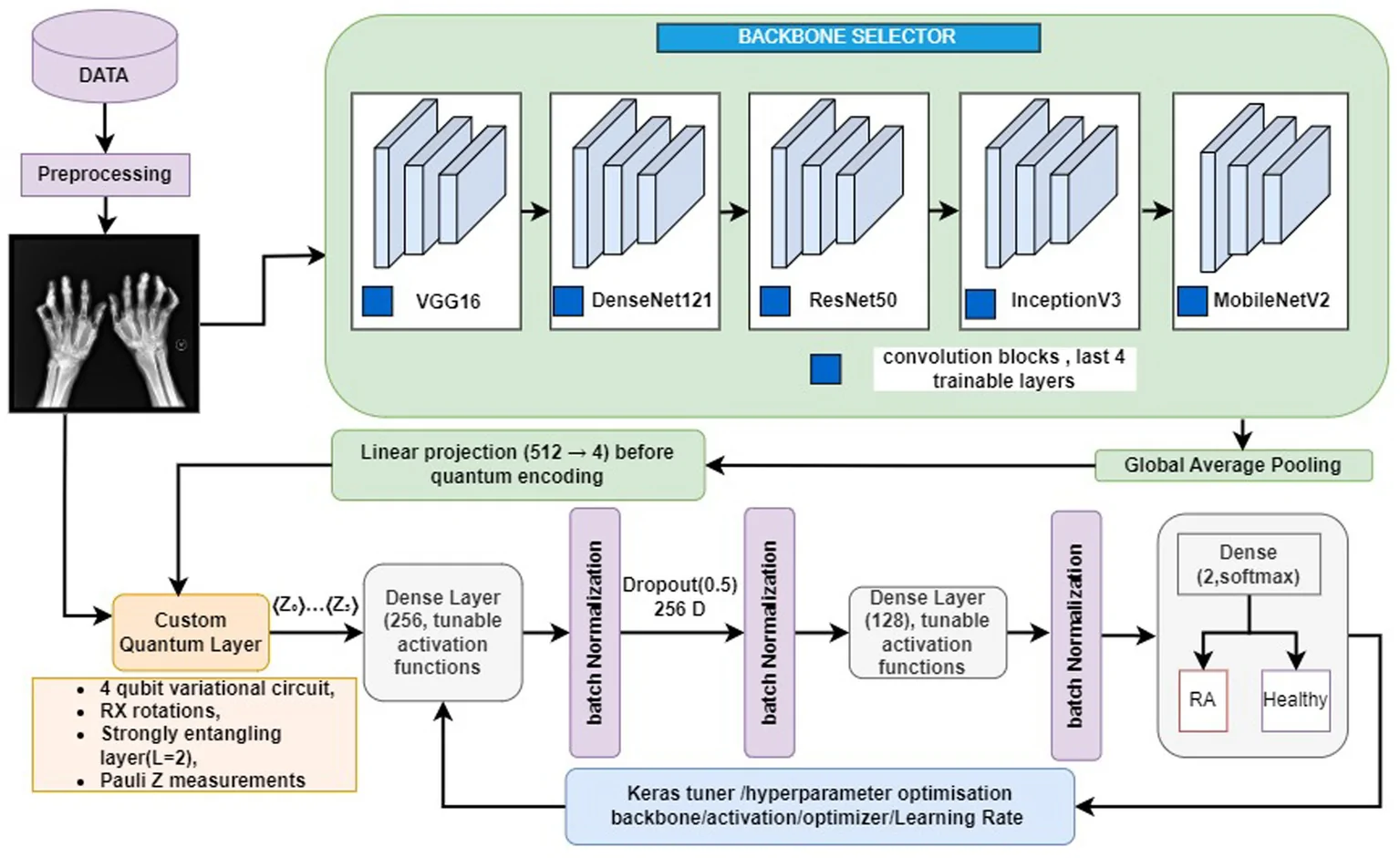
System architecture of the proposed HP-QCNN comprises (i) feature extraction using pretrained CNN backbones, (ii) global average pooling (GAP) followed by a learnable linear projection to a 4-dimensional vector, (iii) RX-based angle embedding into a 4-qubit quantum circuit, (iv) a StronglyEntanglingLayers (SEL) template with L = 2 layers, and (v) a fully connected classical classifier. Stratified data splitting keeps class proportions the same, while class-weighted loss helps address class imbalance.

A Hybrid Pretrained Quantum Convolutional Neural Network (HP-QCNN) is proposed for the automated classification of rheumatoid arthritis in hand X-ray images. In this method, nonlinear feature representation and class discrimination are enhanced by integrating transfer-learning-based feature extraction and variational quantum circuits. Quantum-based transformations to the data, variational quantum circuits, enhance the model’s ability to distinguish between classes. The architecture diagram for the HP-QCNN is depicted in Figure 1. At first, the input radiographs are preprocessed and then passed through a pretrained backbone to extract deep features. Then the resulting 512-dimensional feature vector is linearly projected to a 4-dimensional representation compatible with the 4-qubit quantum circuit. The projected features are then encoded using R_X_-based angle embedding and processed by a variational quantum circuit implemented using PennyLane’s Strongly Entangling Layers template with two layers. The measured expectation values are finally forwarded to the classical dense classification head to predict healthy versus RA-affected classes.

### 3.1 Hand X-ray image preprocessing

**Figure 2:**
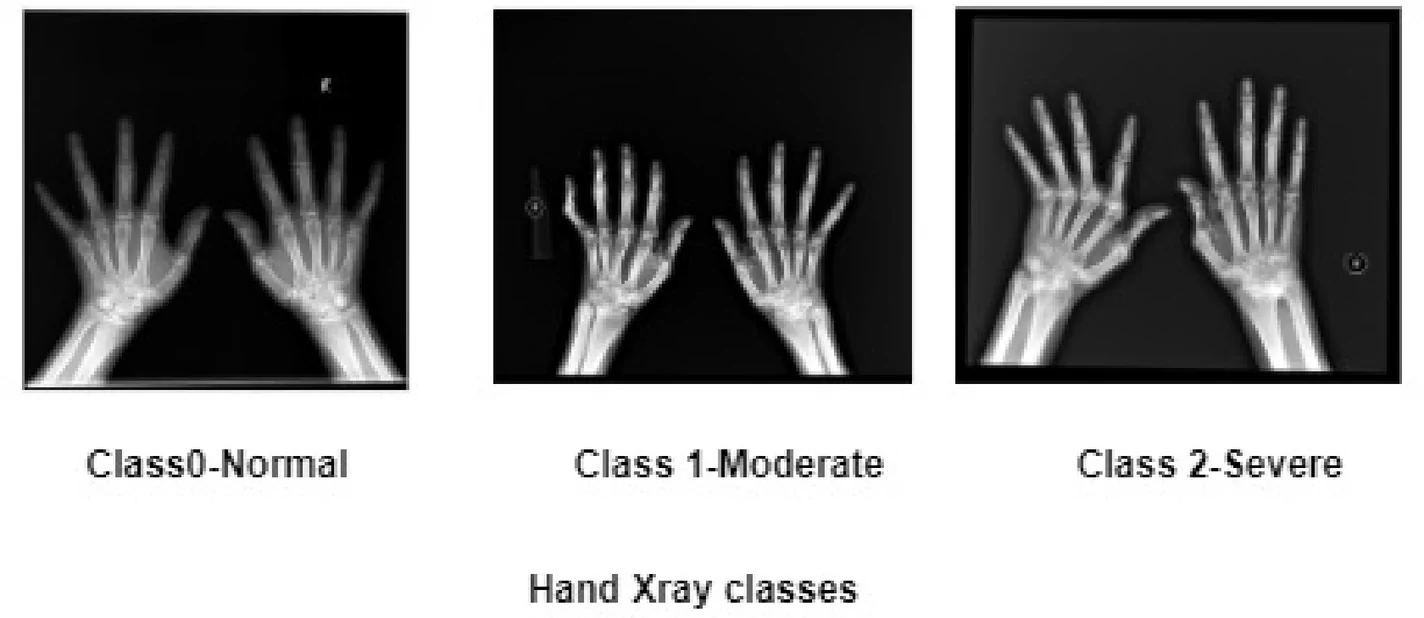
Different types of classes: Hand X-ray.

In preprocessing, the hand X-ray images are resized to 224 × 224 × 3 to ensure compatibility with pretrained convolutional neural networks (CNNs). As part of the preprocessing, intensity normalization and data augmentation (rotation, flipping, and scaling) are used to improve the generalization of the results and avoid overfitting. To preserve the class distribution of rheumatoid arthritis and healthy cases across all splits, the dataset (Wang et al., 2022) is divided into training, validation, and test sets using stratified sampling. The given dataset is split into three different classes based on the modified total sharp score (van der Heijde, 2000), where the overall hand x-ray scores from 0 to 5 are considered healthy, and are mapped to label class 0, scores ranging between 5 and 75 are considered as Moderate, and those who had scores higher than 75 are considered as severe. To align with the binary classification strategy, healthy remained as class 0, and moderate and severe classes were combined as affected, since both have bone erosion and joint space narrowing, which were mapped to class 1, as depicted in Table 3. The three classes of Hand X-ray images are shown in Figure 2. The main objective of our model is to evaluate the efficiency of HP-QCNN in terms of discriminative capability by separating healthy and affected hand X-ray images.

**Table 3:** Mapping of hand X-ray images grade with class labels based on MTSS score.

Grade	Description	Class label	Modified label
Healthy	No bone erosion and joint space narrowing, and mild or near-healthy mTSS < 5	0	0
Moderate	Significant bone erosion, and joint space narrowing. 5 < mTSS < 75	1	1
Severe	Complete bone erosion and joint deformity. mTSS > 75	2	1

### 3.2 CNN backbone selection and feature extraction

As illustrated in the architecture diagram, the preprocessed hand X-ray image bone erosion and joint space narrowing features are extracted using various pretrained networks, such as VGG16 (Simonyan and Zisserman, 2015), DenseNet121 (Huang et al., 2018), ResNet50 (He et al., 2016), InceptionV3 (Szegedy et al., 2016), and MobileNetV2 (Sandler et al., 2018), which utilize the visual knowledge from ImageNet weights. In these pretrained CNN models, the remaining layers, except for the last four trainable layers, are frozen to balance representational power and computational efficiency. In these pretrained networks, VGG16 performs better, which makes it the backbone of the HP-QCNN model. After this, the VGG16-extracted spatial features are converted into a fixed-length 512-dimensional feature vector using a global average pooling layer denoted as $z_{i}\in {\mathbb{R}}^{512}$ , which preserves salient feature information through reducing parameters and mitigates the overfitting.

### 3.3 Linear projection

The high-dimensional feature vector obtained after global average pooling is projected to a 4-dimensional vector using a learnable fully connected (Bengio et al., 2013) dense layer without an activation function, which is defined as


$${f_{i}}^{\prime }=Wz_{i}+b,\text{where}\;z_{i}\in {\mathbb{R}}^{512},W\in {\mathbb{R}}^{4\times 512}\;\text{and}\;b\in {\mathbb{R}}^{4}.$$ (1)

Where $z_{i}$ is the output features from VGG16, W is the trainable projection matrix and b is a bias vector.

This projection is trained jointly with the model to retain the most discriminative features to match the 4-qubit angle encoding.

### 3.4 Feature scaling

Before angle encoding, features are normalized to a bounded interval [0,π ] to ensure proper rotation angles and prevent information loss due to the periodicity of the $R_{X}$ rotation gate which is mentioned below in Equation 2.


$${\tilde{f}}_{i,q}=\pi \frac{f_{i,q}^{\prime }-\min({f_{i}}^{\prime })}{\max({f_{i}}^{\prime })-\min({f_{i}}^{\prime })+\in },$$ (2)

Where i is the sample index, *q* = 0,…,3; feature/quantum index.

$f_{i,q}^{\prime }$ projected feature with respect to the qth qubit of the ith input image.

${\tilde{f}}_{i,q}\;$ —normalized feature and a small constant ∈ (10^−8^) is added to avoid division by zero error, which is used to stabilize the normalization process without significantly altering the scaled feature value.

### 3.5 Variational quantum circuit

**Figure 3:**
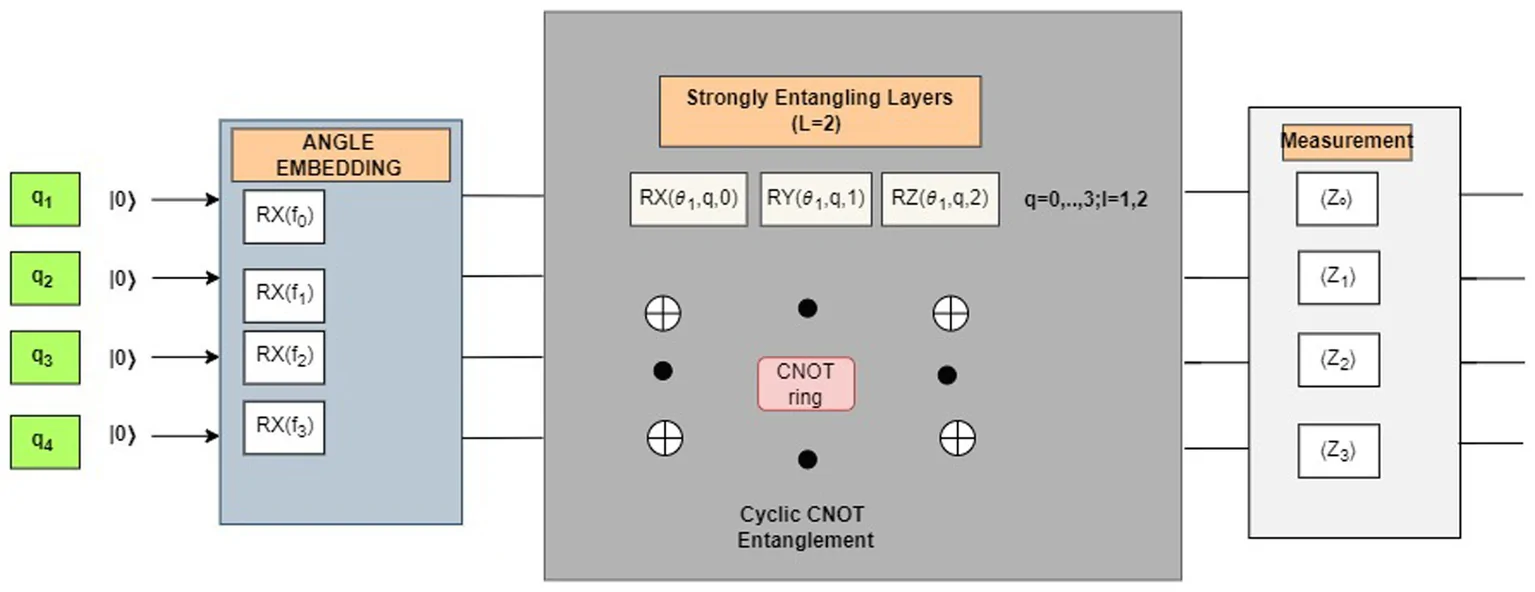
Schematic representation of a 4-qubit variational quantum circuit.

A schematic representation of the 4-qubit variational quantum circuit utilized in the proposed HP-QCNN for the classification of rheumatoid arthritis is depicted in Figure 3. R_X_-based angle embedding is used to encode a 4D classical feature vector extracted from VGG16, which is represented in Equation 3, where each feature is encoded as the rotation angle of an R_X_ gate applied to the corresponding qubit initialized in the ∣0⟩ state. The variational component of the circuit is implemented using a Strongly Entangling Layers (SEL) template with L = 2 layers. In each layer, parameterized single-qubit rotations R_X_, R_Y_, and R_Z_ are applied to all qubits, followed by a cyclic CNOT entanglement pattern that introduces correlations among qubits, as described in Equations 4–7. To produce a 4-dimensional quantum feature vector, the expectation values of Pauli-Z operators are measured on each qubit mentioned in Equation 8. This vector is then fed into the classical classification head. The variational circuit uses two layers, four qubits, and three rotation gates per qubit in each layer for a total of 24 parameters, i.e., $\theta \in {\mathbb{R}}^{2\times 4\times 3}$ .

The classical features are encoded into the quantum circuit using R_X_-based angle encoding (Schuld and Killoran, 2019). The input feature consists of four classical features, and the initial state is $|0\rangle^{⊗4}$ . Before encoding, the features are normalized to a bounded interval [0,π ] compatible with $R_{X}$ and the encoded state is mentioned below in Equation 3.


$$|\Psi_{\mathrm{enc},i}\rangle =|\Psi_{\mathrm{enc},i}\rangle =({⊗}_{q=0}^{3}R_{X}({\tilde{f}}_{i,q}))|0\rangle^{⊗4}$$ (3)

where ${\tilde{f}}_{i,q}$ normalized features.

The variational circuit is implemented using the SEL template with a parameter tensor


$$\theta \in {\mathbb{R}}^{L\times n_{q}\times 3},$$ (4)

where L = 2 is the number of strongly entangling layers, n_q_ = 4 is the number of qubits, resulting in 24 parameters.

For each layer *l* = *1*,…, L, the unity operation is as follows,


$$U_{\mathrm{SEL}}^{\left(l\right)}(\theta^{\left(l\right)})=({⊗}_{n_{q}-1}^{q=0}R_{X}(\theta_{l,q,0})R_{Y}(\theta_{l,q,1})R_{Z}(\theta_{l,q,2}))U_{\text{ring}}$$ (5)

where


$$U_{\text{ring}}=\mathrm{CNO}T_{0\to 1}\mathrm{CNO}T_{1\to 2}\mathrm{CNO}T_{2\to 3}\mathrm{CNO}T_{3\to 0}$$

the full unitary is


$$U(\theta )=\prod_{l=1}^{L}U_{\mathrm{SEL}}^{\left(l\right)}(\theta^{\left(l\right)})$$ (6)

and the final state is


$$|\Psi_{i}\rangle =U(\theta )|\Psi_{\mathrm{enc},i}\rangle$$ (7)

For each qubit q, the Pauli-Z expectation value of the q^th^ qubit of the i^th^ input sample is measured as.


$${\hat{z}}_{i,q}=\langle \Psi_{i}|Z_{q}|\Psi_{i}\rangle ,\text{where}\;{\hat{z}}_{i,q}\in [-1,1],q=0,1,2,3\;$$ (8)

$$\text{forming}\;{\hat{z}}_{i}=({\hat{z}}_{i,0},{\hat{z}}_{i,1},{\hat{z}}_{i,2},{\hat{z}}_{i,3})\in {\mathbb{R}}^{4}$$ (9)

forming the feature vector, as shown in Equation (9), which is used to capture the non-linear, entangled correlations of RA.

### 3.6 Hyperparameter optimization and classification

The quantum feature vector is sent to a classical neural network for final classification. This head has a dense layer with 256 units and activation functions that can be tuned. Batch normalization and dropout (rate = 0.5) for regularization, A second dense layer with 128 units and similar activation choices, Batch normalization followed by a final dense layer with 2 units and SoftMax activation, which gives probabilities for RA and healthy classes. Model optimization was performed using a Keras Tuner–based hyperparameter search, as shown in the diagram. The tuner looks at combinations of CNN backbone selection, activation functions ReLU, LeakyReLU, Mish (Misra, 2020), Swish (Ramachandran et al., 2018), Tanh, Adaptive Mish (Misra, 2020), Optimizers (Adam, Rmsprop, SGD) and learning rates (ReduceLROnPlateau scheduler with factor 2 and patience 5). The model utilized Early stopping with patience 20, sparse categorical loss functions, and calculated class weights to avoid overfitting.

### 3.7 Complexity and parameter efficiency analysis

The proposed Hp-QCNN algorithm is summarized in Table 4. The proposed HP-QCNN framework first preprocesses hand X-ray images through resizing, normalization, stratified data splitting, and class-weight computation. Features are extracted using a pretrained VGG16 backbone with Global Average Pooling and linearly projected to a 4-dimensional vector suitable for quantum processing. The projected features are normalized to [0,*π*], encoded into a 4-qubit quantum circuit using R_X_-based angle encoding, and transformed using a StronglyEntanglingLayers template with L = 2. Pauli-Z expectation values are measured to form a quantum feature vector, which is finally classified using a fully connected classification head optimized through Keras Tuner-based hyperparameter tuning.

**Table 4:** Hybrid pretrained quantum convolutional neural network for Rheumatoid arthritis classification algorithm.

Input: $D_{s}={\left\{(x_{i},y_{i})\right\}}_{i=1}^{N},$ where $x_{i}$ is i^th^ input hand X-ray image, Output: $y_{i}\in \{0,1\}$ where 0-Healty,1-RA Affected, N- No. of samples
1. Given an image, Image in dataset D_s_, $\text{Image}\prime_{i}$ =Resize ( ${\text{Image}}_{i}$ , (224,224)), where, $\text{Image}\prime_{i}$ denotes resized image. 2. $\text{Image}\prime \prime_{i}$ = $\text{Image}\prime_{i}/255$ , where $\text{Image}\prime \prime_{i}$ is the normalized image of each pixel by pixel. 3. Partition $D_{s}$ into training, validation, and test sets using stratified *sampling*, preserving RA/Healthy proportions. 4. Compute class weights $w_{c=}\frac{N}{2N_{c}}$ c ∈{0,1} , where N- total no. of samples, $N_{c}$ - number of samples in class c. 5. Feature extraction using VGG16, for each $\text{Image}\prime \prime_{i}$ $z_{i}=\mathrm{VGG}16(x_{i}),z_{i}\in {\mathbb{R}}^{d},d=512$ , where $z_{i}$ High-dimensional feature vector from VGG16. 6. Apply global average pooling to obtain a fixed-length feature vector and linearly project features $f_{i}^{\prime }=Wz_{i}+b$ , where $z_{i}\in {\mathbb{R}}^{512}$ , $W\in {\mathbb{R}}^{4\times 512}$ and $b\in {\mathbb{R}}^{4}$ 7. Normalize features using ${\tilde{f}}_{i,q}=\pi \frac{f_{i,q}^{\prime }-\min(f_{i}^{\prime })}{\max(f_{i}^{\prime })-\min(f_{i}^{\prime })+\in }$ 8. Encoding classical features using angle encoding $|\Psi_{\mathrm{enc},i}\rangle =({⊗}_{q=0}^{3}\;\;R_{X}({\tilde{f}}_{i,q}))|0\rangle^{⊗4}$ *, where* ${\tilde{f}}_{i,q}$ normalized features. 9. Apply strongly entangling layers $\theta \in {\mathbb{R}}^{L\times n_{q}\times 3}$ , (L = 2, n_q_ = 4). 9.1. For each layer *l* = *1*,., L, the unitary operation is $U_{\mathrm{SEL}}^{\left(l\right)}(\theta^{\left(l\right)})=({⊗}_{q=0}^{n_{q}-1}\;R_{X}(\theta_{l,q,0})\;\;R_{Y}(\theta_{l,q,1})R_{Z}(\theta_{l,q,2}))U_{\text{ring}}$ where $U_{\text{ring}}=\mathrm{CNO}T_{0\to 1}\mathrm{CNO}T_{1\to 2}\mathrm{CNO}T_{2\to 3}\mathrm{CNO}T_{3\to 0}$ 9.2. The full unitary is $U(\theta )=\prod_{l=1}^{L}U_{\mathrm{SEL}}^{\left(l\right)}(\theta^{\left(l\right)})\;$ and the final state is $|\Psi_{i}\rangle =U(\theta )|\Psi_{\mathrm{enc},i}\rangle$ 9.3. Apply cyclic entanglement between qubits to get total trainable parameters as ∣θ∣ = $3Ln_{q}$ =24. 10. For each qubit q, measured Pauli-Z expectation values as ${\hat{z}}_{i,q}=\Psi_{i}\|Z_{q}\|\Psi_{i},\text{where}\;{\hat{z}}_{i,q}\in [-1,1]\;$ , q = 0,1,2,3., 11. ${\hat{z}}_{i}$ = ( ${\hat{z}}_{i,0},{\hat{z}}_{i,1},{\hat{z}}_{i,2},{\hat{z}}_{i,3})\in {\mathbb{R}}^{4}$ 12. Apply a nonlinear transformation to perform classification, ${\hat{y}}_{i}$ $\to \text{Classifier}({\hat{z}}_{i})$ , where ${\hat{y}}_{i}$ predicted labels. 13. Tune the model using various hyperparameters in the case of activation, optimizer, and learning rate using keras tuner.

From the computational time complexity perspective, the training time cost is given in Equation (10),


$$O(N(F_{\mathrm{CNN}}+Ln_{q}C)),$$ (10)

where N denotes the number of training samples, *F_CNN_* denotes the computational cost of CNN-based feature extraction, *L* denotes the number of strongly entangling layers, *n_q_* denotes the number of qubits, and *C* denotes the gate-evaluation cost per qubit per layer in the simulator, which reflects the parameter shift evaluation and feature extraction.

The forwarding inference needs a single quantum pass with the following time complexity $O(F_{\mathrm{CNN}}+Ln_{q})$ which has a small overhead because of a minimal parameter count in the fully connected layer.

The efficiency of the proposed HP-QCNN has been proven by comparing the parameter counts between the dense layer and quantum layer, as mentioned in Table 5, where feature dimension transformations are performed using trainable parameters, which is the influential factor for time complexity calculations. In this table, it is clearly evident that the classical pipeline needs 2052 parameters, whereas the quantum layer needs only 24 parameters. The enhanced feature representation in Hilbert space (Schuld and Killoran, 2019), along with a limited tunable parameter, makes HP-QCNN an efficient and more generalized model in RA hand X-ray classification.

**Table 5:** Parameter comparison between classical dense layer and quantum layer.

Layer	Data dimensions	Parameters	Transformation mechanism
Classical dense layer	512 → 4	4×512+4=2052	Affine projection & activation
Quantum layer	4 → 4	2×4×3=24	Quantum rotations and entanglement

## 4 Experimental setup

### 4.1 Dataset description

The work used a dataset of 3,818 (Wang et al., 2022) hand X-rays that were taken for research and labelled according to Modified Total Sharp Scores (mTSS), which range from 0 to 264. The dataset was split into three classes based on mTSS values. A healthy class that has scores up to 5, which shows no signs of bone erosion and joint space narrowing. Moderate class scores lie between 6 and 75, which shows early erosions and minimal joint space narrowing in x-ray, which indicates the disease is getting worse and the person is having trouble with their daily activities. The third class is derived with the mTSS score greater than 75 and shows high joint space narrowing and heavier bone erosion, which leads to irreversible joint damage. The dataset was split into 2,672 training set images, 382 validation set images, and 764 test set images for model training, tuning the parameters, and unbiased performance evaluation. As the dataset split was image-level rather than patient-level, certain limitations may arise, such as data leakage, if multiple images of the same patient are contained in both the training and validation sets. This will be addressed in the future by using the anonymized dataset with a patient-level split.

### 4.2 Training setup

During the experiment, the initial learning rate was set at 1 × 10^−3^, and the rate was dynamically decreased using the ReduceLROnPlateau scheduler (factor: 0.2; patience: 5 epochs: 50). To avoid overfitting, early stopping with patience (15 epochs) restored the best weights. Calculated class weights addressed class imbalance. The loss function was Sparse Categorical Cross-Entropy, and the evaluation metrics were classification accuracy, balanced accuracy, AUC-ROC curve, precision-recall curve, Matthews Correlation Coefficient, Cohen’s Kappa, and Brier Score Loss. A maximum of 50 epochs was trained. In all experiments, 64 batches were used. Validation-based tuning and performance monitoring optimized batch size, learning rate schedule, and quantum circuit configuration. The table, which can be utilized for reproducibility, is stated below in Table 6.

**Table 6:** Experimental configuration of the proposed HP-QCNN.

Category	Configuration
Environment	Python, TensorFlow, Keras, PennyLane
Backbone & projection	VGG16, last 4 layers trainable, GAP, 512 → 4 projection
Quantum layer	4-qubit VQC, RX encoding, SEL (L = 2), Pauli-Z measurement
Classification	Dense classifier, ReLU, RMSprop, LR = 0.01
Training setup	Sparse Categorical Cross-Entropy, batch size = 64, 50 epochs, ReduceLROnPlateau, Early Stopping, class weights, Keras Tuner

## 5 Result and discussion

### 5.1 Evaluation metrics

The proposed model was evaluated using standard metrics, as defined in Equations (11)–(18). The highest ROC-AUC of HP-QCNN was 95.9%, which shows the model’s effective classification ability, demonstrating the quantum advantage.


$$\text{Accuracy}=\frac{TP_{\mathrm{RA}}+TN_{\mathrm{RA}}\;}{TP_{\mathrm{RA}}+TN_{\mathrm{RA}}+FP_{\mathrm{RA}}+FN_{\mathrm{RA}}}$$ (11)

$$\text{Precision}=\frac{TP_{\mathrm{RA}}}{TP_{\mathrm{RA}}+FP_{\mathrm{RA}}}$$ (12)

$$\;\text{Recall}=\frac{TP_{\mathrm{RA}}}{TP_{\mathrm{RA}}+FN_{\mathrm{RA}}}$$ (13)

$$F1-\text{score}=2.(\frac{\text{precisio}n_{\mathrm{RA}}.\text{recal}l_{\mathrm{RA}}}{\text{precisio}n_{\mathrm{RA}}+\text{recal}l_{\mathrm{RA}}})$$ (14)

$$\text{Specificity}(H)=\frac{TN_{\mathrm{RA}}}{TN_{\mathrm{RA}}+FP_{\mathrm{RA}}}$$ (15)

$$\text{Balanced Accuracy}=\frac{\text{recal}l_{\mathrm{RA}}+\text{Specificity}(H)}{2}$$ (16)

$$\mathrm{MCC}=\frac{TP_{\mathrm{RA}}\times TN_{\mathrm{RA}}-FP_{\mathrm{RA}}\times FN_{\mathrm{RA}}}{\sqrt{\begin{array}{l} \left(TP_{\mathrm{RA}}+FN_{\mathrm{RA}})(TP_{\mathrm{RA}}+FP_{\mathrm{RA}}\right)\\ \left(TN_{\mathrm{RA}}+FN_{\mathrm{RA}})(TN_{\mathrm{RA}}+FP_{\mathrm{RA}}\right) \end{array}}}$$ (17)

$${\text{Cohen}}^{’}s\;\text{Kappa}\;k=\frac{p_{0-p_{e}}}{1-p_{e}}$$ (18)

HP-QCNN demonstrates effective performance across various metrics. The model yields an accuracy as 92.41%, and precision, recall, and f1-score are around 92%, sensitivity as 92.41%, and specificity as 96.63%. Additionally, the proposed method yields 85.19% balanced accuracy, which is robust against a 623:141 class imbalance.0.7382 MCC and 0.7362 Cohen’s Kappa both indicate substantial agreement (>0.70), 0.9015 Fowlkes-Mallows index shows near-perfect precision-recall balance, 0.9591 ROC-AUC shows excellent discrimination among classes, 0.8718 PR-AUC proves that the models work well for any clinical probability (0.3, 0.5, 0.7, 0.9) thresholds for imbalanced detection, 0.0633 Brier score, which is less than 0.1, showed the model is well-calibrated, and 80.58 DOR showed 2.4 × baseline clinical superiority.

**Figure 4:**
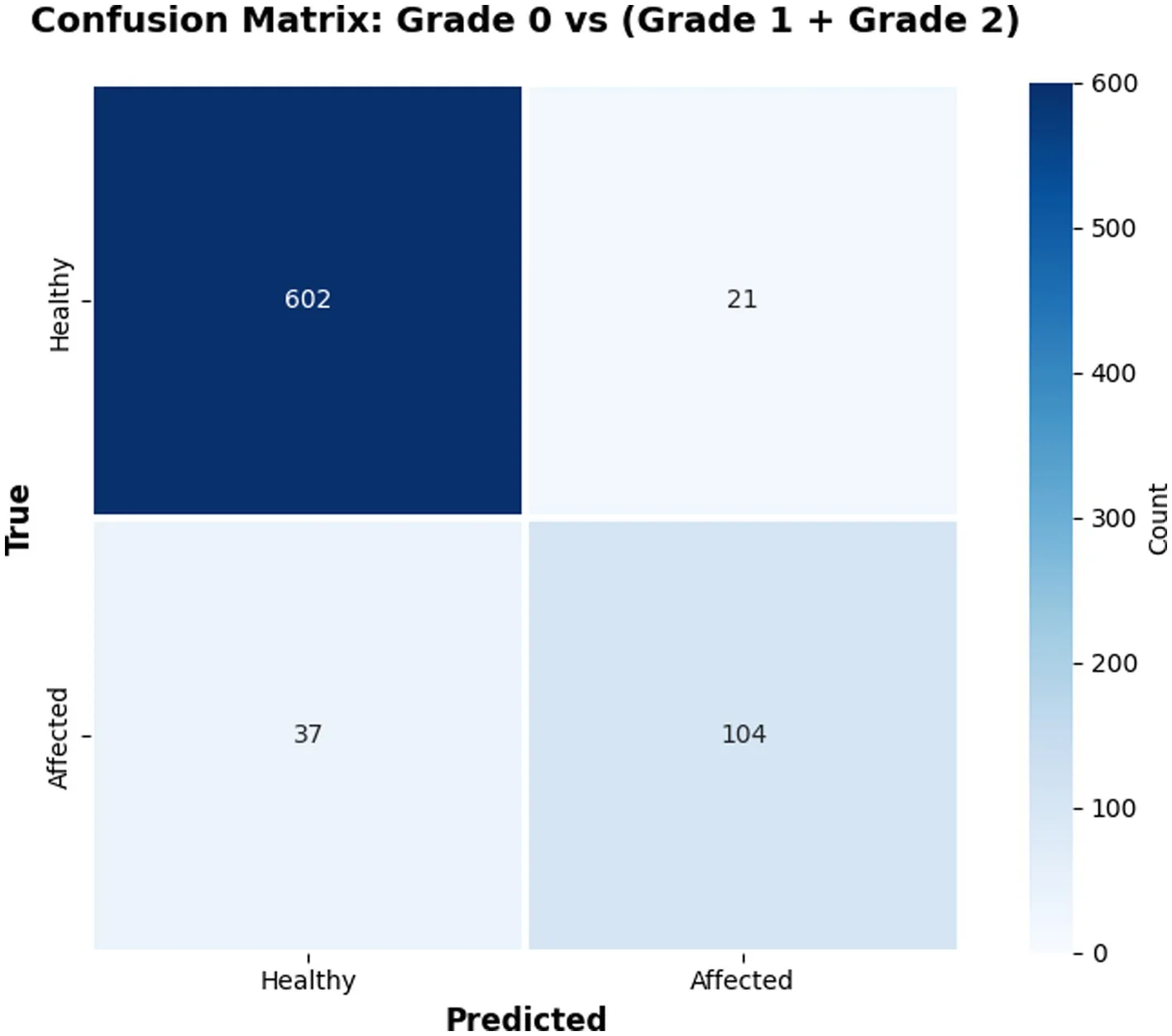
Confusion matrix of HP-QCNN.

The confusion matrix of the HP-QCNN is depicted in Figure 4. It illustrates the relationship between the true and predicted outputs, demonstrating the balanced performance of the classification. The results, with high true positive rates and low misclassification rates, show the model’s effective generalization.

**Figure 5:**
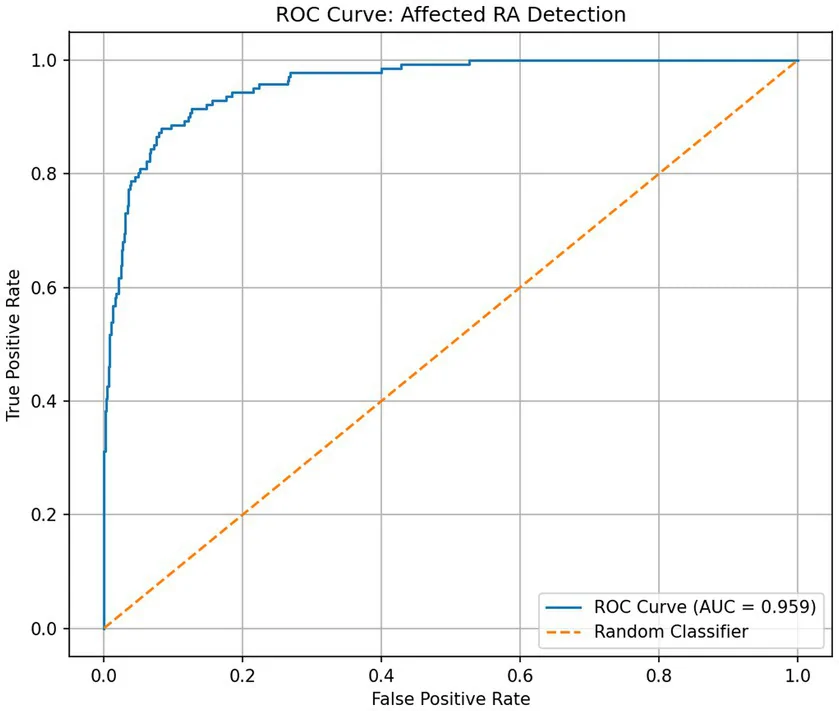
ROC- curve for the affected RA detection.

The AUC-ROC curve of the proposed HP-QCNN is depicted in Figure 5, which demonstrates effective classification ability with a value of 95.9%.

**Figure 6:**
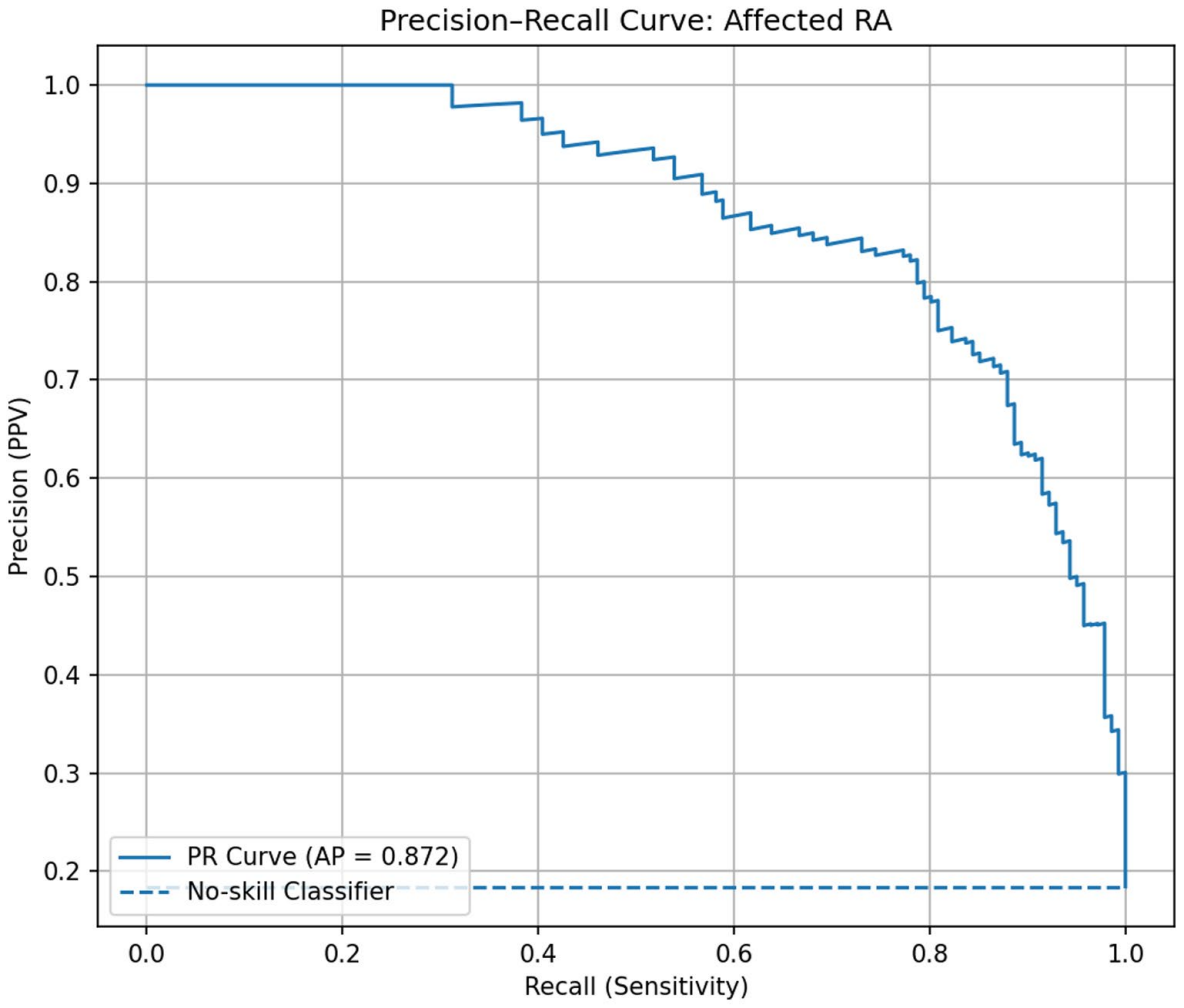
Precision-Recall curve of HP-QCNN.

Figure 6 depicts the Precision Recall curve of HP-QCNN, which shows 0.872, proving that the HP-QCNN model works well at any clinical cutoff thresholds, such as conservative 0.9 or sensitive 0.3.

**Figure 7:**
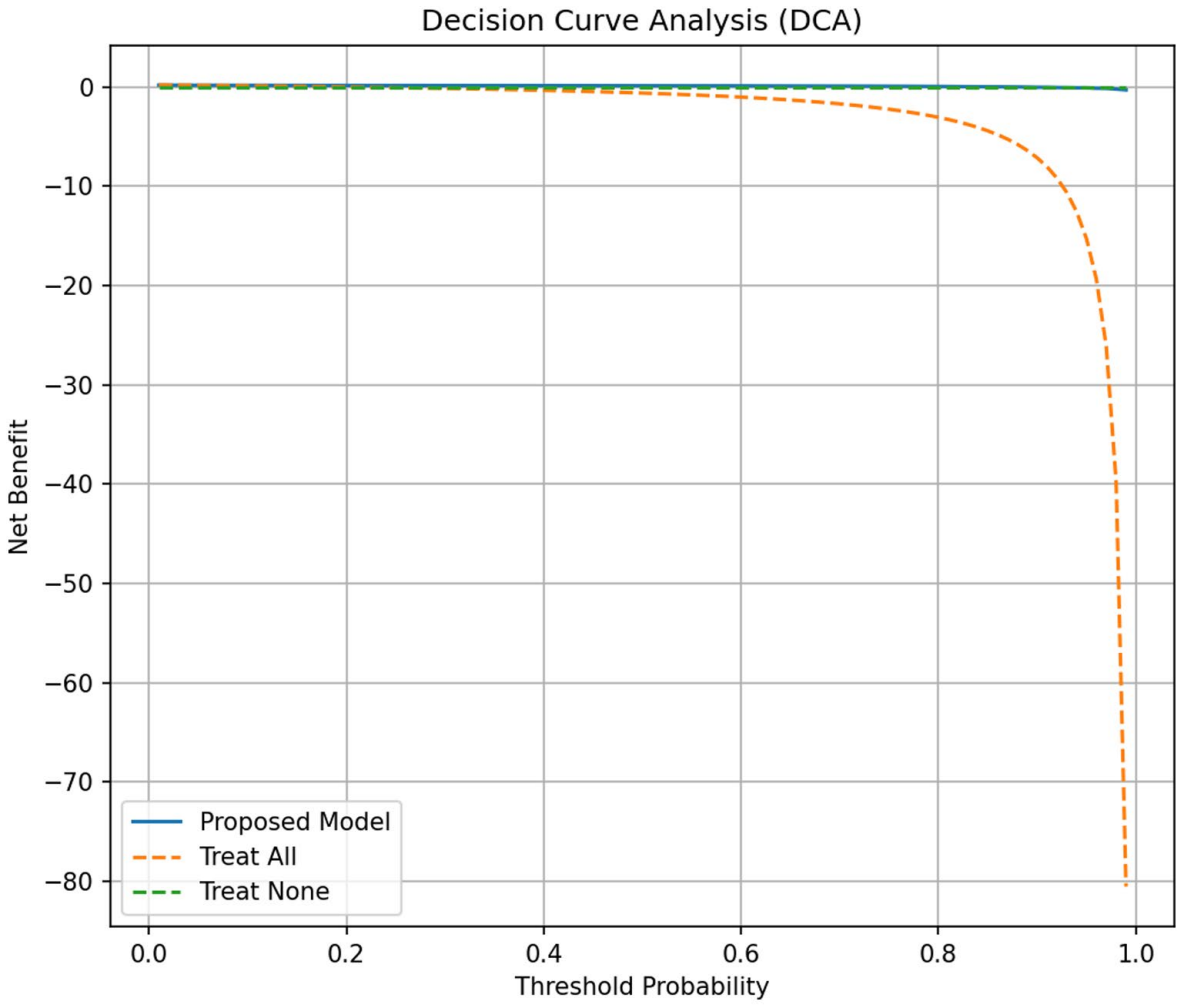
Decision curve analysis of HP-QCNN.

The Decision Curve Analysis graph depicted in Figure 7 has been constructed to weigh the true positives against false positives yield the maximum net benefit where the true positives outweigh false alarms at realistic RA screening thresholds.

### 5.2 Comparative analysis

**Figure 8:**
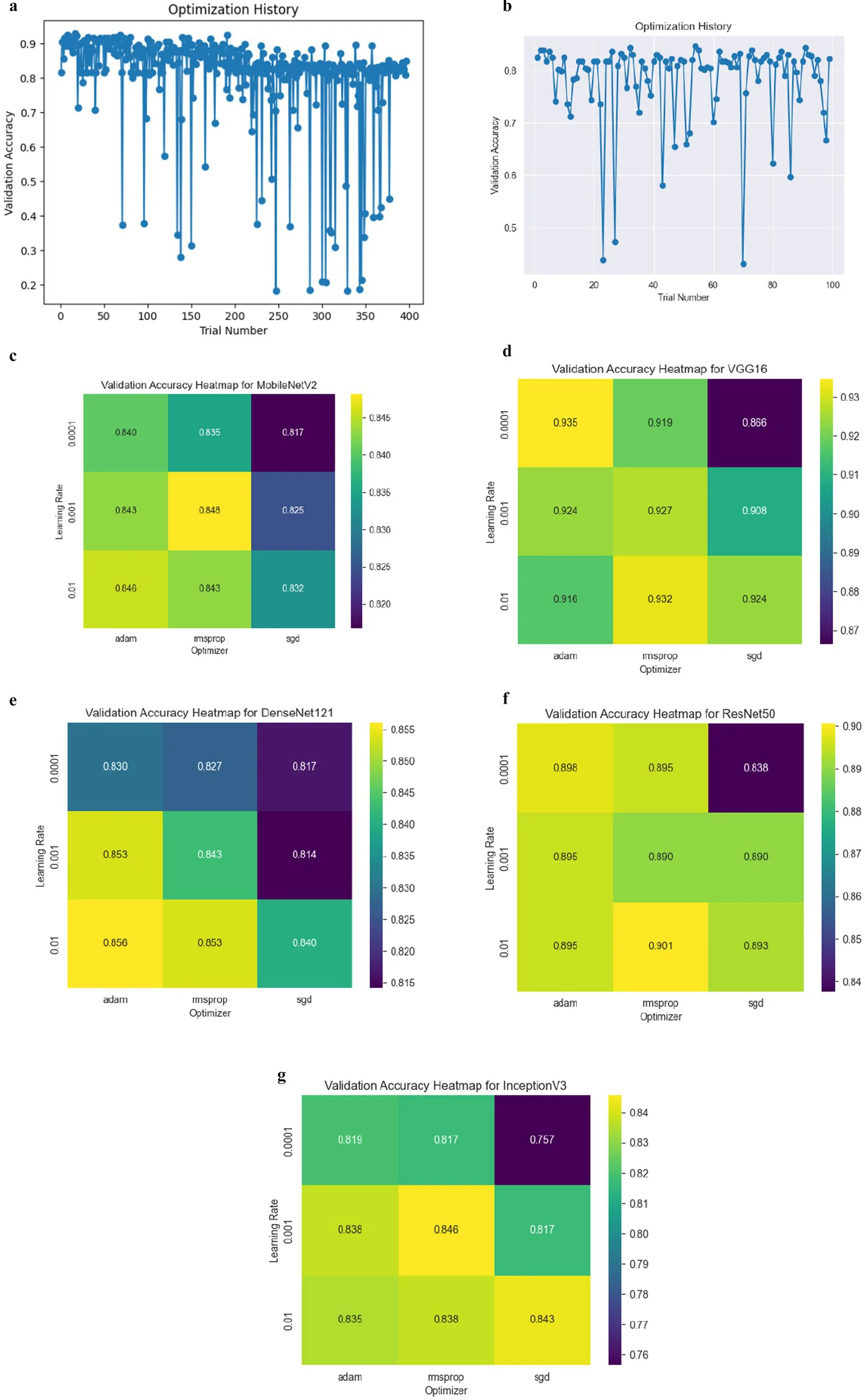
**(a)** VGG16, DenseNet121, ResNet 50, InceptionV3 optimization history. **(b)** MobileNetv2 optimization history. **(c)** MobileNetV2 validation accuracy heatmap. **(d)** VGG16 validation accuracy heatmap. **(e)** DenseNet121 validation accuracy heatmap. **(f)** ResNet50 validation accuracy heatmap. **(g)** InceptionV3 validation accuracy heatmap.

The proposed HP-QCNN efficiency is evaluated by conducting comparative analysis with other backbones such as DenseNet121, VGG16, ResNet50, MobileNetV2, and InceptionV3, along with various optimizers such as SGD, Adam, RMSprop, and different activation functions (Swish, Mish, ReLU, LeakyReLU, Adaptive Mish, and Tanh) for different learning rates. The top-performing combinations for all backbones are listed in Table 7. Keras tuner has been utilized for this hyperband tuning. Figures 8a–g shows the optimization history and backbone-wise heatmap for validation accuracy visualization.

**Table 7:** Top performing combinations.

Backbone	Testing accuracy score	Optimal Config (activation &optimizer& LR)
DenseNet121	90.84%	Swish, Adam, lr = 0.01
MobileNetV2	90.52%	Swish, RMSprop, lr = 0.01
ResNet50	87.17%	Swish, RMSprop, lr = 0.0001
InceptionV3	85.07%	ReLU, RMSprop, lr = 0.01
VGG16	92.41%	ReLU, RMSprop, lr = 0.01

Table 8 shows a comparative analysis of HP-QCNN with recent works that have been done in rheumatoid arthritis prediction using hand X-rays and hand thermal images. The table shows that the HP-QCNN outperformed VGG 8, CNN with VGG16 transfer, RANet, RA-XTNET, and YOLOv4+VGG16, with the accuracy of 74, 83, 88, 90.7, 90.60%, respectively. The proposed HP-QCNN also outperformed the existing model with other metrics such as specificity, precision, ROC-AUC, balanced accuracy, Mathew Correlation Coefficient, and Cohen’s kappa statistics, by handling data imbalance, and shows better class separation capability.

**Table 8:** Comparative analysis between proposed HP-QCNN and recent works.

Source	Model	Accuracy	Specificity	Precision	ROC-AUC	Balanced accuracy	MCC	C-kappa
Rohrbach et al. (2019)	VGG16 transfer	83%	–	–	–	83%	–	56.6%
Üreten and Maraş (2022)	YOLOv4 with VGG16 transfer	90.7%	88.7%	86.3%	–	–	–	–
RA-XTNet Kesavapillai et al. (2024)	RA–XTNET	90.60%	–	–	95.5%	82%	65%	–
Rajesh et al. (2024)	RANet	88%	87%	87.67%	–	–	–	–
VGG RA (Cai et al., 2025)	VGG8	74%	88%	83%	81%	–	–	–
Proposed model	HP–QCNN	92.41%	92.4%	92.4%	95.9%	85.19%	73.82%	73.62%

### 5.3 Ablation study

The proposed HP-QCNN performance has been compared with the baseline model (VGG16) to analyze the ablation study, which is depicted in Table 9.

**Table 9:** Ablation study between HP-QCNN and baseline.

Model	Accuracy	Precision	Recall	F1-score	ROC-AUC	PR-AUC	Specificity	Diagnostic odds ratio
VGG16 Baseline	86.13%	66.95%	81.54%	73.25%	0.9266	0.7739	86.68	33.38
HP-QCNN	92.41%	92.4%	92.4%	92.41%	0.9591	0.8718	96.63	80.58

**Figure 9:**
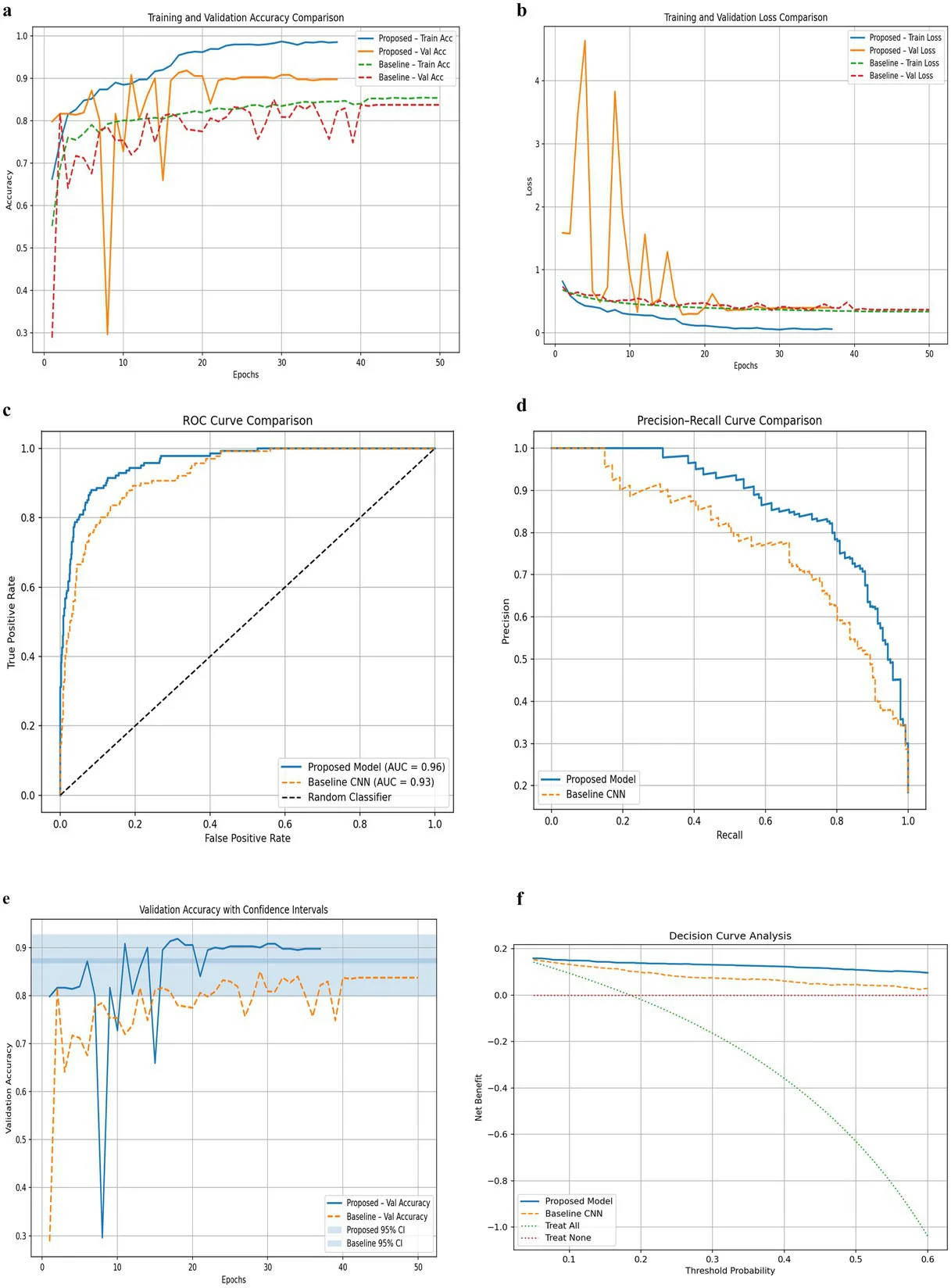
**(a)** Training and validation accuracy comparison. **(b)** Training and validation loss comparison. **(c)** ROC curve comparison. **(d)** PR-curve comparison. **(e)** Validation accuracy with confidence intervals comparison. **(f)** Decision Curve Analysis comparison.

The Ablation study shows the proposed HP-QCNN used quantum advantage through PennyLane 4-qubit variational circuits, achieving 92.41% test accuracy, balanced accuracy of 85.19%, and ROC-AUC of 0.9591, which surpassed the VGG16 baseline by more than 6.2% accuracy and the highest diagnostic odds ratio with 80.58. 10-fold cross-validation confirms MCC as 0.7382 and Cohen’s Kappa value as 0.7362. Quantum ablation yields +6.2% accuracy, which leads to a quantum-clinical translation benchmark for RA classification. The comparative analysis graph between the proposed HP-QCNN and baseline in terms of Training Accuracy, Validation accuracy, validation loss, ROC curve, PR-Curve, and DOC curve is depicted in Figures 9a–f. The training and validation learning curves show a mild overfitting after epoch 15, which is based on a scenario in which training accuracy continues to increase while validation loss varies. This outcome is likely attributable to the constrained parameter capacity of the 4-qubit variational quantum circuit and the remaining class imbalance, which may render optimization more susceptible to samples from the minority class. The proposed HP-QCNN employs early stopping, class-weighted optimization, dropout regularization, and validation-based learning rate scheduling to reduce these effects, improving training stability and generalization. Figures 9c–f shows the comparative analysis between Baseline versus HP-QCNN in terms of ROC, PR, validation accuracy with confidence intervals, and decision curve, demonstrating that the proposed HP-QCNN outperformed the baseline model through improved feature representation and performance stability obtained from the hybrid quantum layer.

**Figure 10:**
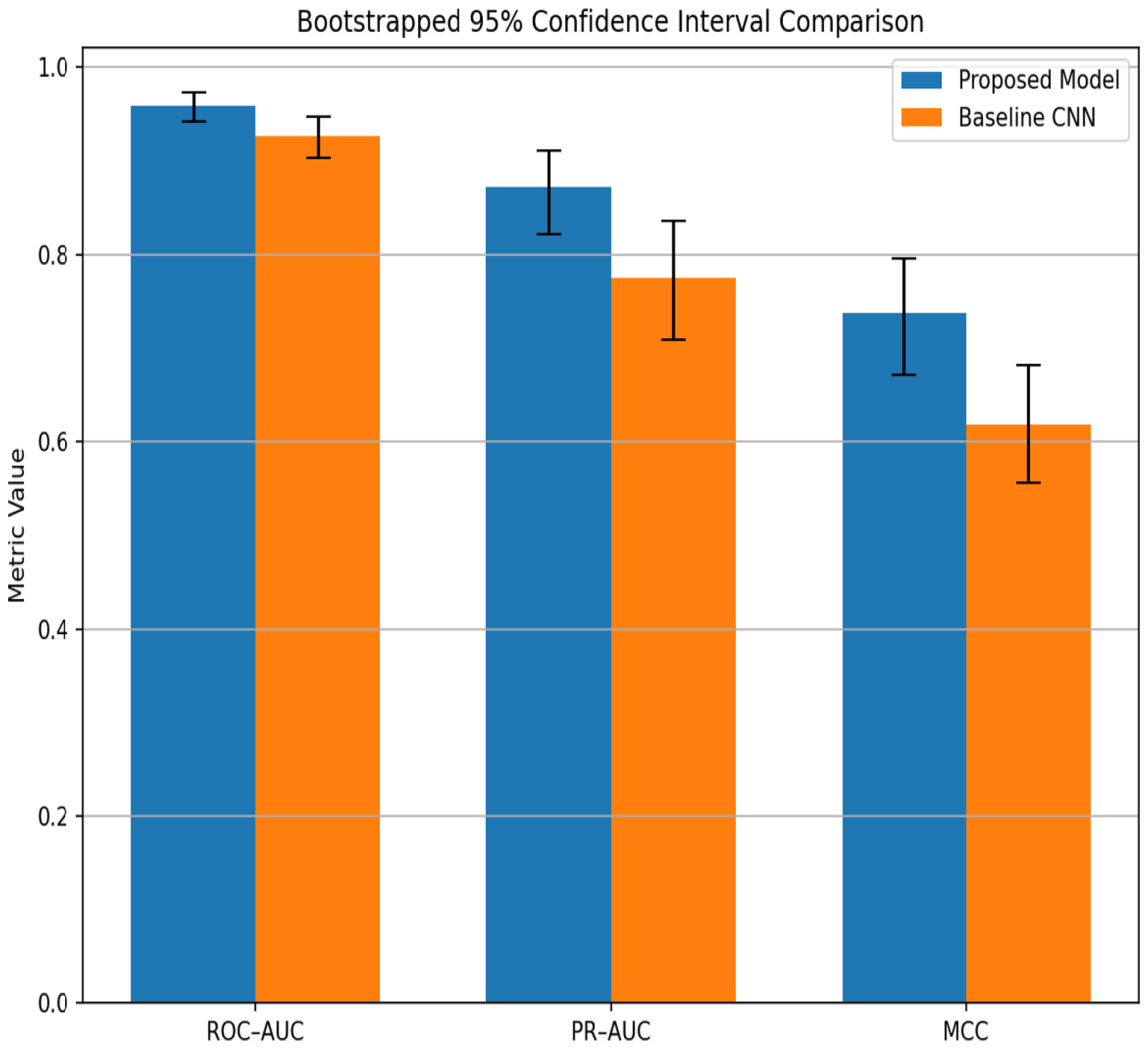
Performance evaluation comparison between baseline and Proposed HP-QCNN.

Figure 10 the bootstrapped 95% CIs confirm that HP-QCNN performs better than baseline VGG16 in all the performance metrics, ROC-AUC, PR-AUC, and MCC across 1,000 resamples with *n* = 764 test samples that provide evidence of quantum-enhanced performance for clinical RA classification. Figures 8a,b shows the hyperparameter optimization history of VGG16, DenseNet121, ResNet50, InceptionV3, MobileNetV2, and Figures 8c–g depicts the validation accuracy heatmaps, which demonstrate the sensitivity of the model’s accuracy to optimizers and activation functions of various backbones. The heatmaps show that the VGG16 with the ReLU activation function and RMSprop achieved the highest validation accuracy.

### 5.4 Computational efficiency

The proposed HP-QCNN classification efficiency has been evaluated using the inference time required to classify a single hand X-ray with a batch size of one. The average inference time of HP-QCNN is 120.55 ms per image, and the throughput is roughly 8.30 images per second. The minimum and maximum inference times are 86.01 ms and 495.47 ms, respectively, with a standard deviation of 49.65 ms. These results demonstrate that the proposed HP-QCNN model is computationally efficient and practical for near-real-time clinical RA screening.

## 6 Conclusion

HP-QCNN represents a hybrid architecture that utilises quantum advantage through a 4-qubit variational circuit, angle embedding, and strongly entangled layers for RA hand X-ray classification. It achieved 92.41% accuracy, 95.91% ROC-AUC, 96.63% specificity, and 85.19% balanced accuracy, which outperformed existing models such as VGG 8, VGG16 transfer, RANet, RA-XTNET, and YOLOv4+VGG16 using standard X-ray only. The model outperformed the existing model in terms of agreement metrics such as MCC and Cohen’s kappa, with the values over 73%, while other models rely on standard metrics and omit important metrics such as ROC -AUC, MCC, Cohen’s kappa, and balanced accuracy. While decision curve analysis confirmed that the model net benefited across various clinical cutoffs and non-overlapping bootstrapping CIs, validation showed a *p*-value under 0.01 for HP-QCNN, confirming the quantum advantage over baseline VGG16 across various performance metrics. The parameter count reduction with enhanced feature representation improves model generalization in rheumatoid arthritis classification. The model performed well with a simulated quantum environment, which needs to be deployed on original quantum hardware without limitations. At the primary level for the prediction of RA, the proposed method combined moderate and severe as a single class to perform binary classification. Even though the model performed well, in the context of clinical practice, it needs different treatment plans for moderate and severe cases. To address this limitation, the secondary work focused on exploring multi-level classification to capture early signs of RA through RA progression prediction in the future. In addition, future work will involve collaboration with expert rheumatologists and radiologists to perform joint annotation, severity grading, and validation of the proposed HP-QCNN’s practical applicability in real-world systems. Moreover, it will be evaluated using multi-centre datasets collected from various hospitals and diverse X-ray imaging systems for broader clinical generalizability and will also focus on incorporating an explainable quantum mechanism to enhance interpretability.

## Data Availability

Publicly available datasets were analyzed in this study. This data can be found at: https://drive.google.com/drive/folders/1O11ROJypqyBksrfYqTIjdXxdc6MBao27?usp=sharing.
